# Effects of Art Therapy emotion Expression of Students

**DOI:** 10.1192/j.eurpsy.2026.11684

**Published:** 2026-07-31

**Authors:** L. Utaş Akhan, F. Kiras

**Affiliations:** 1 Mental Health, Bandırma Onyedi Eylül University, Bandırma; 2 Mental Health, Balıkesir Kepsut Public Hospital, Balıkesir, Türkiye

## Abstract

**Introduction:**

The study population consisted of 74 fourth-year students studying in the Nursing Department of Bandırma Onyedi Eylül University’s Faculty of Health Sciences during the 2024-2025 academic year.

**Objectives:**

First, students were interviewed and informed about the purpose and importance of the research. Written consent was obtained from students who agreed to participate in the study, and data collection forms were administered by the researchers using a face-to-face interview technique. The art therapy course was conducted two hours per week for 12 weeks with students in the experimental group. The course was conducted in the central classrooms of Bandırma Onyedi Eylül University by the faculty member responsible, Latife UTAŞ AKHAN, who holds a doctoral degree in art therapy and an art therapy practice certificate. In the first week of the art therapy course, students were introduced to the course and provided with information about its scope.

**Methods:**

The relevant course for the fourth year is offered as an elective in the “Art Therapy” course. The minimum data size for the study was determined as 40 students, with at least 20 in the experimental and control groups, a 95% confidence interval, an effect size of 0.8, and a theoretical power of 80%.Accordingly, an experimental area was created for those who enrolled in an elective art therapy course based on minimal dimensions and refused to participate. A control group was selected on a voluntary basis to be homogeneous with the experimental group in terms of gender (as gender is a significant factor in the variables to be examined) and scale scores.Data were collected using an Introductory Information Form and the Abbreviated Expressed Emotion Scale.

**Results:**

**Table 1.** Table 1. Long description.

Characteristics	Intervention Group n (%)	Control Group n (%)	χ²	p-value
Gender			0.073	0.787
Female	16	16		
Male	4	4		
Age			0.217	0.641
18–22 years	17	17		
≥22 years	3	3		
Economic status			0.189	0.910
Good	7	8		
Moderate	10	11		
Bad	3	1		
Family history of psychiatric disorder			0.558	0.455
No	18	17		
Yes	2	3

**Conclusions:**

The study found an increase in motivational sub-tension in the emotion regulation variable, an increase in positive emotional sub-relationships in the happiness variable, and an increase in positive emotional changes and closeness warmth in the experimental groups.

**Disclosure of Interest:**

None Declared

## Long descriptions

### Table 1. Long description

The table consists of five columns: Characteristics, Intervention Group n (%), Control Group n (%), chi-squared, and p-value.

* Gender: The Intervention Group has 16 females and 4 males. The Control Group has 16 females and 4 males. The chi-squared value is 0.073 and the p-value is 0.787.

* Age: For the 18 to 22 years category, both groups have 17 participants. For the greater than or equal to 22 years category, both groups have 3 participants. The chi-squared value is 0.217 and the p-value is 0.641.

* Economic status: In the Intervention Group, 7 are Good, 10 are Moderate, and 3 are Bad. In the Control Group, 8 are Good, 11 are Moderate, and 1 is Bad. The chi-squared value is 0.189 and the p-value is 0.910.

* Family history of psychiatric disorder: In the Intervention Group, 18 answered No and 2 answered Yes. In the Control Group, 17 answered No and 3 answered Yes. The chi-squared value is 0.558 and the p-value is 0.455.

Navigate back to Table 1..

